# Crystal structure of 3-(3-oxo-2,3,4,4a,5,6-hexa­hydro­benzo[*h*]cinnolin-2-yl)propionic acid

**DOI:** 10.1107/S1600536814019850

**Published:** 2014-09-06

**Authors:** Fiorella Meneghetti, Daniela Masciocchi, Arianna Gelain, Stefania Villa

**Affiliations:** a Department of Pharmaceutical Sciences, University of Milano, via L. Mangiagalli, 25, 20133-Milano, Italy

**Keywords:** crystal structure, pyridazinone moiety, stat3 inhibitor

## Abstract

The asymmetric unit of the title compound, C_15_H_16_N_2_O_3_, contains two independent mol­ecules, which present a different conformation of the carb­oxy­lic acid side chain [C—C—C—OH torsion angles = 65.3 (7) and −170.1 (5)°]. In both mol­ecules, the di­hydro­pyridazinone ring adopts a geometry inter­mediate between a twisted-boat and a half-chair conformation, while the central six-membered ring is almost in a half-boat conformation. In the crystal, mol­ecules are linked by O—H⋯O_k_ (k = ketone) hydrogen bonds, generating [01-1] chains. Aromatic π–π stacking contacts between the benzene and the di­hydro­pyridazinone rings [centroid–centroid distance [3.879 (9) Å] are also observed.

## Related literature   

For background to the bioactivity of pyridazinone derivatives, see: Masciocchi *et al.* (2013 ▶). For structural and mol­ecular modeling studies, see: Toma *et al.* (1990 ▶). For the chemistry of pyridazinone derivatives, see: Costantino *et al.* (1996 ▶).
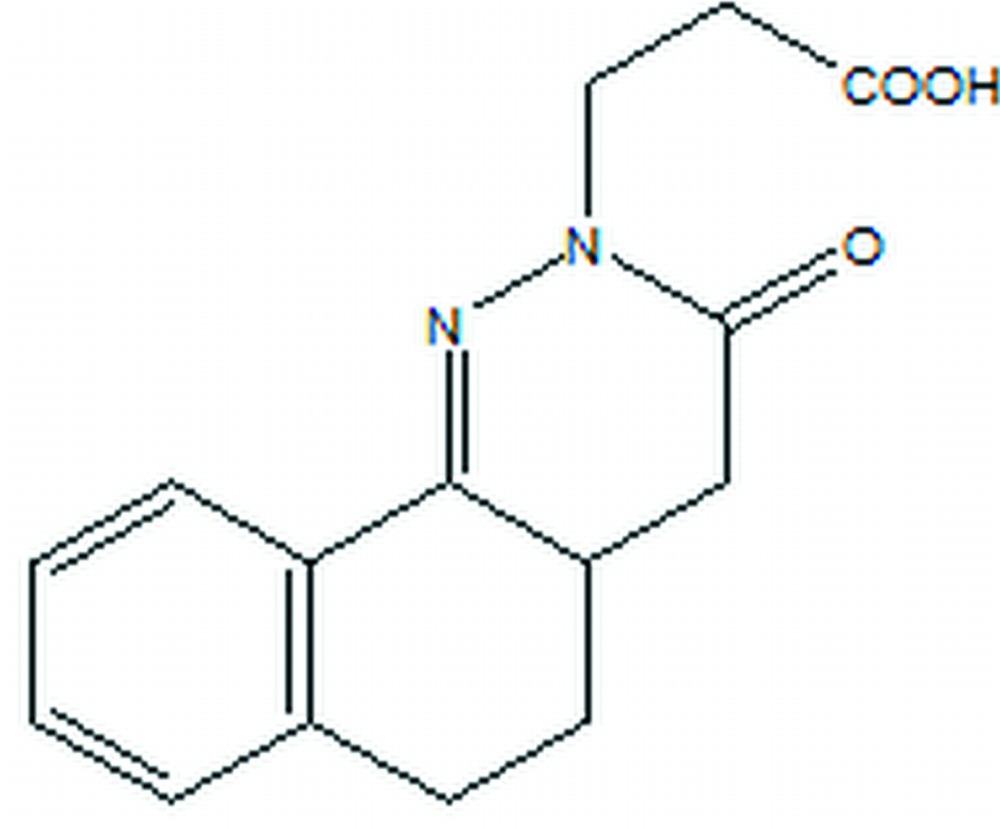


## Experimental   

### Crystal data  

C_15_H_16_N_2_O_3_
*M* *_r_* = 272.3
Triclinic, 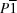

*a* = 11.217 (4) Å
*b* = 11.668 (4) Å
*c* = 12.110 (4) Å
α = 79.22 (1)°
β = 64.62 (1)°
γ = 68.630 (9)°
*V* = 1332.6 (8) Å^3^
*Z* = 4
Mo *K*α radiation
μ = 0.10 mm^−1^
*T* = 293 K
0.65 × 0.45 × 0.40 mm

### Data collection  

Enraf–Nonius TurboCAD-4 diffractometer
5412 measured reflections
4682 independent reflections
1412 reflections with *I* > 2σ(*I*)
*R*_int_ = 0.081
3 standard reflections every 120 min intensity decay: 9%

### Refinement  

*R*[*F* ^2^ > 2σ(*F* ^2^)] = 0.059
*wR*(*F* ^2^) = 0.205
*S* = 0.92
4682 reflections
368 parameters
H atoms treated by a mixture of independent and constrained refinement
Δρ_max_ = 0.28 e Å^−3^
Δρ_min_ = −0.22 e Å^−3^

### 

Data collection: *CAD-4 EXPRESS* (Enraf–Nonius, 1994 ▶); cell refinement: *CAD-4 EXPRESS*; data reduction: *XCAD4* (Harms & Wocadlo, 1995 ▶); program(s) used to solve structure: *SIR92* (Altomare *et al.*, 1994 ▶); program(s) used to refine structure: *SHELXL97* (Sheldrick, 2008 ▶); molecular graphics: *ORTEP-3 for Windows* (Farrugia, 2012 ▶); software used to prepare material for publication: *WinGX* (Farrugia, 2012 ▶).

## Supplementary Material

Crystal structure: contains datablock(s) global, I. DOI: 10.1107/S1600536814019850/hb7233sup1.cif

Structure factors: contains datablock(s) I. DOI: 10.1107/S1600536814019850/hb7233Isup2.hkl

Supporting information file. DOI: 10.1107/S1600536814019850/hb7233Isup3.cml

**Fig. 1.**
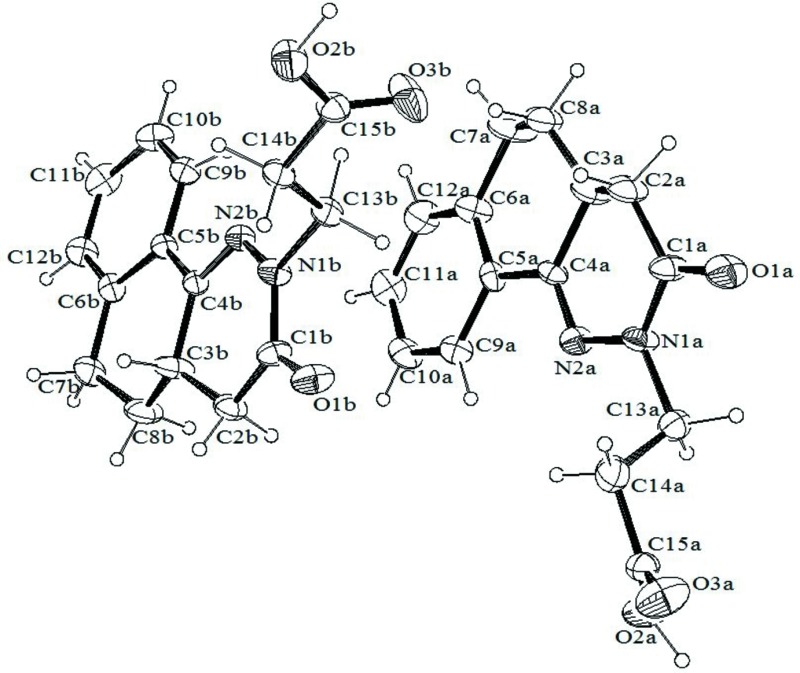
: The molecular structure of the asymmetric unit of the title compound, showing displacement ellipsoids for non-H atoms at the 40% probability level.

. DOI: 10.1107/S1600536814019850/hb7233fig1.tif
The mol­ecular structure of the asymmetric unit of the title compound, showing displacement ellipsoids for non-H atoms at the 40% probability level.

**Fig. 2.**
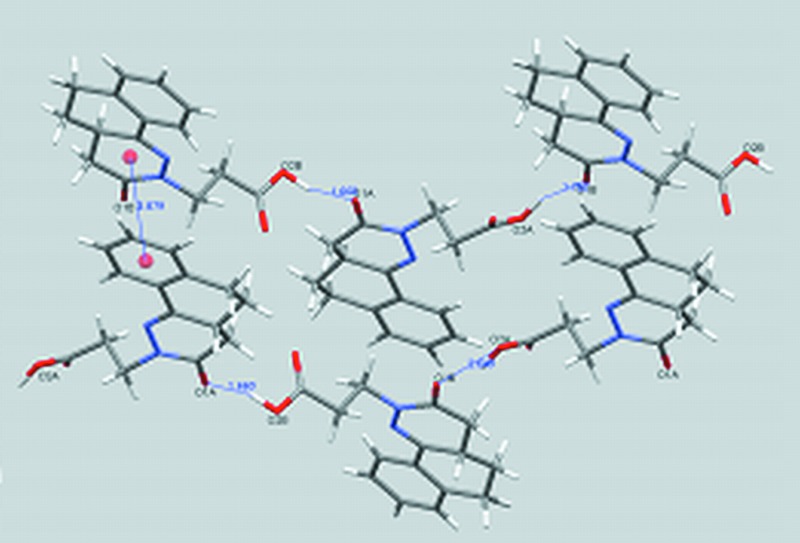
: Intermolecular interactions of the title compound. Hydrogen bonds are shown as dashed lines.

. DOI: 10.1107/S1600536814019850/hb7233fig2.tif
Inter­molecular inter­actions of the title compound. Hydrogen bonds are shown as dashed lines.

CCDC reference: 874435

Additional supporting information:  crystallographic information; 3D view; checkCIF report

## Figures and Tables

**Table 1:** Hydrogen-bond geometry (Å, °)

*D*—H⋯*A*	*D*—H	H⋯*A*	*D*⋯*A*	*D*—H⋯*A*
O2*A*—H2*A*⋯O1*B*	0.92 (7)	1.78 (7)	2.651 (6)	158 (6)
O2*B*—H2*B*⋯O1*A*	0.90 (6)	1.75 (6)	2.598 (7)	157 (5)

## supplementary crystallographic information

### S1. Structural commentary

In our previous researches focused on the discovery of new inhibitors targeting aberrant STAT3 signaling for the treatment of human cancers, we evidenced that several pyridazinone derivatives were able to inter­fer within the STAT3 pathway (Masciocchi *et al.*, 2013). As the size of the central ring plays a main role in determining the conformational properties for this class of compounds, we investigated the extent of planarity of the phenyl with respect to the other cycles by crystallographic analysis determining the molecular structure of the title compound. The asymmetric unit of the title compound (Fig. 1) is characterized by two crystallographically independent molecules (*a* and *b*). The values obtained for the bond length and angles of the two independent molecules are in accordance with each other, whilst at the same time presenting a different conformation of the carb­oxy­lic chain linked to N1. This difference is best evidenced by the torsion angles N2—N1—C13—C14 of 76 (1)°[-100 (1)°] and O2—C15—C14—C13 of 65 (1)°[-170 (1)°] (the values in the square brackets refer to the *b* labeled molecule). The tricyclic skeleton of the compound consists of three fused rings slightly twisted with respect to each other. The dihedral angles between their least-square planes α (C1/N1/N2/C4/C3/C2), β (C3/C4/C5/C6/C7/C8) and γ (C5/C6/C12/C11/C10/C9) are: α-β = 5.0 (1)°[3.0 (1)°], α-γ = 11.5 (1)°[10.3 (1)°], β-γ = 6.6 (1)°[7.3 (1)°], respectively. In detail, the di­hydro­pyridazinone ring adopts a geometry inter­mediate between a twisted-boat and a half-chair conformation qu­anti­tatively defined by the parameters QT = 0.321 (6)Å[0.242 (6)Å], φ = -87 (1)°[-83 (1)°] and θ = 113.2 (9)°[114 (1)°], while the central six-membered ring is almost in a half-boat conformation, characterized by the puckering parameters QT = 0.372 (7)Å[0.334 (7)Å], φ = -64 (1)°[-58 (1)°] and θ = 57 (1)°[59 (1)°], with the flap atom C8 out of the best mean plane calculated over the other five carbons by 0.512 (6)Å[0.462 (6)Å]. In the crystal, the *a* and *b* molecules are flattened and lay on planes deviated from that containing the *a* and *c* axes by about 30°. The two conformers inter­act through π-π contacts between the benzene and the di­hydro­pyridazinone rings, at a centroid-centroid distance of 3.879 (9)Å. In addition, *a* and *b* molecules are inter­connected through hydrogen bonds, where the carb­oxy­lic oxygen O2 is donor of a proton to the ketonic oxygen O1 of the partner molecule (Fig. 2). The inter­molecular contacts involve O2a which is linked to a centrosymmetrically related molecule of *b* (O2a—H···O1b(i) at a distance of 1.78 (7)Å and angle of 158 (6)° [symmetry code: (i) -*x*, 1 - *y*, 1 - *z*)] and O2b that hydrogen bonds a molecule related to *a* by a crystallographic inversion centre (O2b–H···O1a(ii) at a distance of 1.75 (6)Å and angle of 157 (5)° [symmetry code:(ii) at -*x*, 2 - *y*, - *z*)].

### S2. Crystallization

After many attempts weakly diffracting yellow prisms were grown by slow evaporation of a 30:70 water/methanol solution.

### S3. Refinement

Crystal data, data collection and structure refinement details are summarized in Table 1. All non-H-atoms were refined anisotropically. The H-atoms positions bonded to heteroatoms were obtained by a close examination of a final difference Fourier, while the remaining ones were introduced at calculated positions and refined with fixed isotropic thermal parameters (1.2 Ueq of the parent atom).

### Crystal data

**Table:**

C_15_H_16_N_2_O_3_	*Z* = 4
*M**_r_* = 272.3	*F*(000) = 576
Triclinic, *P*1	*D*_x_ = 1.357 Mg m^−^^3^
Hall symbol: -P 1	Mo *K*α radiation, λ = 0.71073 Å
*a* = 11.217 (4) Å	Cell parameters from 25 reflections
*b* = 11.668 (4) Å	θ = 9–10°
*c* = 12.110 (4) Å	µ = 0.10 mm^−^^1^
α = 79.22 (1)°	*T* = 293 K
β = 64.62 (1)°	Prism, yellow
γ = 68.630 (9)°	0.65 × 0.45 × 0.40 mm
*V* = 1332.6 (8) Å^3^	

### Data collection

**Table:**

Enraf–Nonius TurboCAD-4 diffractometer	*R*_int_ = 0.081
Radiation source: fine-focus sealed tube	θ_max_ = 25.0°, θ_min_ = 2.1°
Graphite monochromator	*h* = −12→13
non–profiled ω/2θ scans	*k* = −13→13
5412 measured reflections	*l* = −1→14
4682 independent reflections	3 standard reflections every 120 min
1412 reflections with *I* > 2σ(*I*)	intensity decay: 9%

### Refinement

**Table:**

Refinement on *F*^2^	Primary atom site location: structure-invariant direct methods
Least-squares matrix: full	Secondary atom site location: difference Fourier map
*R*[*F*^2^ > 2σ(*F*^2^)] = 0.059	Hydrogen site location: inferred from neighbouring sites
*wR*(*F*^2^) = 0.205	H atoms treated by a mixture of independent and constrained refinement
*S* = 0.92	*w* = 1/[σ^2^(*F*_o_^2^) + (0.0932*P*)^2^] where *P* = (*F*_o_^2^ + 2*F*_c_^2^)/3
4682 reflections	(Δ/σ)_max_ < 0.001
368 parameters	Δρ_max_ = 0.28 e Å^−^^3^
0 restraints	Δρ_min_ = −0.22 e Å^−^^3^

### Special details

**Table:**

Geometry. All e.s.d.'s (except the e.s.d. in the dihedral angle between two l.s. planes) are estimated using the full covariance matrix. The cell e.s.d.'s are taken into account individually in the estimation of e.s.d.'s in distances, angles and torsion angles; correlations between e.s.d.'s in cell parameters are only used when they are defined by crystal symmetry. An approximate (isotropic) treatment of cell e.s.d.'s is used for estimating e.s.d.'s involving l.s. planes.
Refinement. Refinement of *F*^2^ against ALL reflections. The weighted *R*-factor *wR* and goodness of fit *S* are based on *F*^2^, conventional *R*-factors *R* are based on *F*, with *F* set to zero for negative *F*^2^. The threshold expression of *F*^2^ > σ(*F*^2^) is used only for calculating *R*-factors(gt) *etc*. and is not relevant to the choice of reflections for refinement. *R*-factors based on *F*^2^ are statistically about twice as large as those based on *F*, and *R*- factors based on ALL data will be even larger.

### Fractional atomic coordinates and isotropic or equivalent isotropic displacement parameters (Å^2^)

**Table:**

	*x*	*y*	*z*	*U*_iso_*/*U*_eq_	
O1A	0.0119 (4)	0.6619 (4)	0.0134 (4)	0.0688 (13)	
O2A	−0.0208 (4)	0.3563 (4)	0.4257 (4)	0.0671 (14)	
H2A	−0.067 (6)	0.299 (6)	0.451 (6)	0.08 (2)*	
O3A	−0.2262 (5)	0.4797 (4)	0.4325 (5)	0.0882 (16)	
N1A	0.1447 (4)	0.5851 (4)	0.1231 (4)	0.0496 (13)	
N2A	0.2481 (5)	0.5828 (4)	0.1591 (4)	0.0471 (13)	
C1A	0.1087 (6)	0.6616 (5)	0.0356 (6)	0.0530 (17)	
C2A	0.1924 (6)	0.7439 (6)	−0.0252 (6)	0.068 (2)	
H2A1	0.2002	0.7583	−0.1092	0.081*	
H2A2	0.1415	0.8224	0.0140	0.081*	
C3A	0.3308 (6)	0.7041 (6)	−0.0269 (6)	0.0621 (18)	
H3A	0.3821	0.6377	−0.0852	0.075*	
C4A	0.3353 (6)	0.6383 (5)	0.0923 (5)	0.0383 (14)	
C5A	0.4477 (6)	0.6302 (5)	0.1270 (5)	0.0408 (14)	
C6A	0.5553 (5)	0.6744 (5)	0.0508 (5)	0.0520 (17)	
C7A	0.5541 (6)	0.7381 (7)	−0.0711 (6)	0.081 (2)	
H7A1	0.5920	0.8051	−0.0879	0.097*	
H7A2	0.6149	0.6797	−0.1354	0.097*	
C8A	0.4166 (6)	0.7870 (6)	−0.0752 (6)	0.067 (2)	
H8A1	0.4281	0.8098	−0.1595	0.080*	
H8A2	0.3654	0.8618	−0.0294	0.080*	
C9A	0.4508 (6)	0.5720 (5)	0.2382 (5)	0.0484 (16)	
H9A	0.3789	0.5420	0.2913	0.058*	
C10A	0.5593 (6)	0.5589 (5)	0.2699 (6)	0.0576 (18)	
H10A	0.5600	0.5200	0.3442	0.069*	
C11A	0.6657 (6)	0.6025 (6)	0.1934 (7)	0.068 (2)	
H11A	0.7392	0.5929	0.2148	0.081*	
C12A	0.6632 (6)	0.6610 (6)	0.0840 (6)	0.0662 (19)	
H12A	0.7350	0.6917	0.0320	0.079*	
C13A	0.0675 (6)	0.4990 (5)	0.1958 (6)	0.0529 (16)	
H13A	0.1326	0.4197	0.2051	0.063*	
H13B	0.0167	0.4871	0.1534	0.063*	
C14A	−0.0311 (6)	0.5484 (5)	0.3179 (6)	0.0610 (18)	
H14A	−0.0990	0.6255	0.3081	0.073*	
H14B	0.0194	0.5652	0.3576	0.073*	
C15A	−0.1058 (7)	0.4600 (5)	0.3977 (6)	0.0529 (16)	
O1B	0.1349 (4)	0.8168 (3)	0.4536 (4)	0.0599 (12)	
O2B	0.0424 (5)	1.2275 (4)	0.1748 (4)	0.0815 (16)	
H2B	0.038 (6)	1.246 (5)	0.101 (5)	0.070*	
O3B	0.1379 (6)	1.0503 (4)	0.0884 (5)	0.0973 (19)	
N1B	0.3133 (4)	0.8868 (4)	0.3469 (4)	0.0422 (12)	
N2B	0.4445 (4)	0.8964 (4)	0.3189 (4)	0.0374 (11)	
C1B	0.2460 (6)	0.8252 (5)	0.4432 (5)	0.0435 (15)	
C2B	0.3141 (6)	0.7674 (5)	0.5298 (5)	0.0506 (16)	
H2B1	0.2426	0.7792	0.6121	0.061*	
H2B2	0.3534	0.6795	0.5173	0.061*	
C3B	0.4248 (6)	0.8110 (6)	0.5232 (5)	0.0609 (19)	
H3B	0.3709	0.8868	0.5689	0.073*	
C4B	0.4978 (5)	0.8586 (4)	0.3997 (5)	0.0347 (13)	
C5B	0.6346 (5)	0.8694 (4)	0.3670 (5)	0.0355 (14)	
C6B	0.6924 (5)	0.8414 (5)	0.4544 (5)	0.0425 (15)	
C7B	0.6210 (6)	0.7916 (5)	0.5799 (5)	0.0535 (17)	
H7B1	0.6902	0.7264	0.6035	0.064*	
H7B2	0.5783	0.8566	0.6376	0.064*	
C8B	0.5143 (7)	0.7434 (6)	0.5873 (6)	0.069 (2)	
H8B1	0.5608	0.6606	0.5571	0.083*	
H8B2	0.4553	0.7367	0.6731	0.083*	
C9B	0.7096 (5)	0.9085 (5)	0.2506 (5)	0.0453 (15)	
H9B	0.6733	0.9244	0.1912	0.054*	
C10B	0.8366 (6)	0.9246 (5)	0.2198 (6)	0.0570 (18)	
H10B	0.8844	0.9527	0.1418	0.068*	
C11B	0.8898 (6)	0.8975 (5)	0.3091 (6)	0.0593 (19)	
H11B	0.9754	0.9066	0.2904	0.071*	
C12B	0.8196 (6)	0.8580 (5)	0.4235 (6)	0.0489 (16)	
H12B	0.8572	0.8418	0.4820	0.059*	
C13B	0.2663 (5)	0.9335 (5)	0.2464 (5)	0.0463 (15)	
H13C	0.2232	0.8791	0.2377	0.056*	
H13D	0.3459	0.9344	0.1705	0.056*	
C14B	0.1637 (6)	1.0613 (5)	0.2699 (5)	0.0510 (16)	
H14C	0.2069	1.1154	0.2791	0.061*	
H14D	0.0842	1.0601	0.3458	0.061*	
C15B	0.1141 (6)	1.1115 (6)	0.1669 (6)	0.0479 (15)	

### Atomic displacement parameters (Å^2^)

**Table:**

	*U*^11^	*U*^22^	*U*^33^	*U*^12^	*U*^13^	*U*^23^
O1A	0.069 (3)	0.075 (3)	0.081 (3)	−0.027 (2)	−0.057 (3)	0.031 (2)
O2A	0.051 (3)	0.069 (3)	0.083 (3)	−0.025 (3)	−0.037 (3)	0.031 (3)
O3A	0.063 (3)	0.064 (3)	0.130 (5)	−0.018 (3)	−0.044 (3)	0.025 (3)
N1A	0.044 (3)	0.063 (3)	0.046 (3)	−0.024 (3)	−0.025 (3)	0.022 (3)
N2A	0.046 (3)	0.051 (3)	0.057 (3)	−0.017 (3)	−0.037 (3)	0.017 (3)
C1A	0.055 (4)	0.046 (4)	0.055 (4)	−0.016 (3)	−0.028 (4)	0.021 (3)
C2A	0.073 (5)	0.080 (5)	0.065 (5)	−0.035 (4)	−0.049 (4)	0.039 (4)
C3A	0.056 (4)	0.076 (4)	0.052 (4)	−0.021 (4)	−0.031 (4)	0.026 (4)
C4A	0.040 (3)	0.039 (3)	0.032 (3)	−0.008 (3)	−0.019 (3)	0.009 (3)
C5A	0.043 (3)	0.036 (3)	0.043 (4)	0.000 (3)	−0.026 (3)	−0.006 (3)
C6A	0.030 (3)	0.067 (4)	0.048 (4)	−0.011 (3)	−0.014 (3)	0.012 (3)
C7A	0.049 (4)	0.117 (6)	0.060 (5)	−0.039 (4)	−0.013 (4)	0.038 (4)
C8A	0.078 (5)	0.078 (5)	0.055 (4)	−0.041 (4)	−0.036 (4)	0.033 (4)
C9A	0.041 (4)	0.044 (3)	0.049 (4)	−0.002 (3)	−0.020 (3)	0.004 (3)
C10A	0.061 (4)	0.054 (4)	0.059 (5)	−0.001 (3)	−0.042 (4)	0.006 (3)
C11A	0.046 (4)	0.077 (5)	0.081 (6)	−0.008 (4)	−0.032 (4)	−0.010 (4)
C12A	0.044 (4)	0.087 (5)	0.067 (5)	−0.025 (4)	−0.019 (4)	−0.001 (4)
C13A	0.051 (4)	0.050 (4)	0.064 (5)	−0.019 (3)	−0.029 (4)	0.007 (3)
C14A	0.067 (4)	0.056 (4)	0.063 (5)	−0.019 (4)	−0.027 (4)	−0.006 (4)
C15A	0.066 (4)	0.054 (4)	0.052 (4)	−0.030 (4)	−0.033 (4)	0.016 (3)
O1B	0.046 (2)	0.061 (3)	0.078 (3)	−0.023 (2)	−0.036 (2)	0.026 (2)
O2B	0.128 (4)	0.046 (3)	0.085 (4)	0.002 (3)	−0.082 (4)	0.004 (3)
O3B	0.139 (5)	0.066 (3)	0.081 (4)	0.026 (3)	−0.081 (4)	−0.018 (3)
N1B	0.032 (3)	0.052 (3)	0.044 (3)	−0.009 (2)	−0.026 (2)	0.012 (3)
N2B	0.031 (3)	0.038 (3)	0.040 (3)	−0.008 (2)	−0.017 (2)	0.004 (2)
C1B	0.037 (4)	0.039 (3)	0.055 (4)	−0.012 (3)	−0.025 (3)	0.016 (3)
C2B	0.057 (4)	0.060 (4)	0.042 (4)	−0.025 (3)	−0.029 (3)	0.018 (3)
C3B	0.056 (4)	0.079 (5)	0.050 (4)	−0.032 (4)	−0.029 (4)	0.035 (4)
C4B	0.040 (3)	0.028 (3)	0.037 (4)	−0.006 (2)	−0.020 (3)	0.001 (3)
C5B	0.036 (3)	0.034 (3)	0.039 (4)	−0.006 (3)	−0.023 (3)	0.003 (3)
C6B	0.040 (4)	0.038 (3)	0.046 (4)	−0.001 (3)	−0.023 (3)	−0.003 (3)
C7B	0.054 (4)	0.058 (4)	0.053 (4)	−0.010 (3)	−0.036 (3)	0.007 (3)
C8B	0.090 (5)	0.083 (5)	0.055 (5)	−0.047 (4)	−0.047 (4)	0.037 (4)
C9B	0.040 (4)	0.042 (3)	0.047 (4)	−0.003 (3)	−0.023 (3)	0.006 (3)
C10B	0.036 (3)	0.056 (4)	0.062 (5)	−0.012 (3)	−0.011 (3)	0.012 (3)
C11B	0.037 (4)	0.056 (4)	0.085 (6)	−0.010 (3)	−0.031 (4)	0.004 (4)
C12B	0.039 (4)	0.051 (4)	0.058 (5)	−0.003 (3)	−0.032 (4)	0.002 (3)
C13B	0.037 (3)	0.055 (4)	0.046 (4)	−0.009 (3)	−0.023 (3)	0.004 (3)
C14B	0.057 (4)	0.051 (4)	0.047 (4)	−0.011 (3)	−0.032 (3)	0.009 (3)
C15B	0.052 (4)	0.039 (4)	0.049 (4)	−0.005 (3)	−0.026 (3)	0.003 (3)

### Geometric parameters (Å, º)

**Table:**

O1A—C1A	1.225 (6)	O1B—C1B	1.236 (6)
O2A—C15A	1.330 (7)	O2B—C15B	1.295 (7)
O2A—H2A	0.92 (6)	O2B—H2B	0.90 (6)
O3A—C15A	1.174 (6)	O3B—C15B	1.175 (6)
N1A—C1A	1.353 (7)	N1B—C1B	1.332 (6)
N1A—N2A	1.395 (5)	N1B—N2B	1.403 (5)
N1A—C13A	1.477 (6)	N1B—C13B	1.466 (6)
N2A—C4A	1.272 (6)	N2B—C4B	1.287 (6)
C1A—C2A	1.465 (8)	C1B—C2B	1.478 (7)
C2A—C3A	1.441 (7)	C2B—C3B	1.472 (7)
C2A—H2A1	0.9700	C2B—H2B1	0.9700
C2A—H2A2	0.9700	C2B—H2B2	0.9700
C3A—C8A	1.480 (8)	C3B—C8B	1.448 (7)
C3A—C4A	1.515 (7)	C3B—C4B	1.478 (7)
C3A—H3A	0.9800	C3B—H3B	0.9800
C4A—C5A	1.459 (7)	C4B—C5B	1.459 (7)
C5A—C6A	1.377 (7)	C5B—C9B	1.388 (7)
C5A—C9A	1.399 (7)	C5B—C6B	1.397 (7)
C6A—C12A	1.380 (7)	C6B—C12B	1.389 (7)
C6A—C7A	1.526 (8)	C6B—C7B	1.501 (7)
C7A—C8A	1.457 (7)	C7B—C8B	1.461 (7)
C7A—H7A1	0.9700	C7B—H7B1	0.9700
C7A—H7A2	0.9700	C7B—H7B2	0.9700
C8A—H8A1	0.9700	C8B—H8B1	0.9700
C8A—H8A2	0.9700	C8B—H8B2	0.9700
C9A—C10A	1.376 (7)	C9B—C10B	1.384 (7)
C9A—H9A	0.9300	C9B—H9B	0.9300
C10A—C11A	1.365 (8)	C10B—C11B	1.384 (8)
C10A—H10A	0.9300	C10B—H10B	0.9300
C11A—C12A	1.378 (8)	C11B—C12B	1.357 (8)
C11A—H11A	0.9300	C11B—H11B	0.9300
C12A—H12A	0.9300	C12B—H12B	0.9300
C13A—C14A	1.481 (8)	C13B—C14B	1.503 (7)
C13A—H13A	0.9700	C13B—H13C	0.9700
C13A—H13B	0.9700	C13B—H13D	0.9700
C14A—C15A	1.504 (8)	C14B—C15B	1.514 (7)
C14A—H14A	0.9700	C14B—H14C	0.9700
C14A—H14B	0.9700	C14B—H14D	0.9700
			
C15A—O2A—H2A	107 (4)	C15B—O2B—H2B	102 (4)
C1A—N1A—N2A	125.5 (5)	C1B—N1B—N2B	125.6 (4)
C1A—N1A—C13A	120.9 (5)	C1B—N1B—C13B	121.2 (4)
N2A—N1A—C13A	113.5 (4)	N2B—N1B—C13B	112.4 (4)
C4A—N2A—N1A	119.2 (4)	C4B—N2B—N1B	118.6 (4)
O1A—C1A—N1A	121.5 (5)	O1B—C1B—N1B	119.5 (5)
O1A—C1A—C2A	124.3 (5)	O1B—C1B—C2B	124.2 (5)
N1A—C1A—C2A	114.2 (5)	N1B—C1B—C2B	116.2 (5)
C3A—C2A—C1A	117.4 (5)	C3B—C2B—C1B	117.4 (5)
C3A—C2A—H2A1	107.9	C3B—C2B—H2B1	108.0
C1A—C2A—H2A1	107.9	C1B—C2B—H2B1	108.0
C3A—C2A—H2A2	107.9	C3B—C2B—H2B2	108.0
C1A—C2A—H2A2	107.9	C1B—C2B—H2B2	108.0
H2A1—C2A—H2A2	107.2	H2B1—C2B—H2B2	107.2
C2A—C3A—C8A	120.7 (5)	C8B—C3B—C2B	120.4 (5)
C2A—C3A—C4A	111.5 (5)	C8B—C3B—C4B	114.2 (5)
C8A—C3A—C4A	112.6 (5)	C2B—C3B—C4B	112.9 (5)
C2A—C3A—H3A	103.2	C8B—C3B—H3B	101.9
C8A—C3A—H3A	103.2	C2B—C3B—H3B	101.9
C4A—C3A—H3A	103.2	C4B—C3B—H3B	101.9
N2A—C4A—C5A	119.0 (5)	N2B—C4B—C5B	117.1 (5)
N2A—C4A—C3A	121.2 (5)	N2B—C4B—C3B	123.2 (5)
C5A—C4A—C3A	119.6 (5)	C5B—C4B—C3B	119.7 (5)
C6A—C5A—C9A	118.3 (5)	C9B—C5B—C6B	118.4 (5)
C6A—C5A—C4A	121.7 (5)	C9B—C5B—C4B	121.7 (5)
C9A—C5A—C4A	119.9 (5)	C6B—C5B—C4B	119.9 (5)
C5A—C6A—C12A	120.4 (5)	C12B—C6B—C5B	119.1 (5)
C5A—C6A—C7A	119.2 (5)	C12B—C6B—C7B	119.6 (5)
C12A—C6A—C7A	120.4 (6)	C5B—C6B—C7B	121.3 (5)
C8A—C7A—C6A	114.2 (5)	C8B—C7B—C6B	113.1 (5)
C8A—C7A—H7A1	108.7	C8B—C7B—H7B1	109.0
C6A—C7A—H7A1	108.7	C6B—C7B—H7B1	109.0
C8A—C7A—H7A2	108.7	C8B—C7B—H7B2	109.0
C6A—C7A—H7A2	108.7	C6B—C7B—H7B2	109.0
H7A1—C7A—H7A2	107.6	H7B1—C7B—H7B2	107.8
C7A—C8A—C3A	116.2 (5)	C3B—C8B—C7B	118.4 (5)
C7A—C8A—H8A1	108.2	C3B—C8B—H8B1	107.7
C3A—C8A—H8A1	108.2	C7B—C8B—H8B1	107.7
C7A—C8A—H8A2	108.2	C3B—C8B—H8B2	107.7
C3A—C8A—H8A2	108.2	C7B—C8B—H8B2	107.7
H8A1—C8A—H8A2	107.4	H8B1—C8B—H8B2	107.1
C10A—C9A—C5A	120.6 (6)	C10B—C9B—C5B	122.4 (5)
C10A—C9A—H9A	119.7	C10B—C9B—H9B	118.8
C5A—C9A—H9A	119.7	C5B—C9B—H9B	118.8
C11A—C10A—C9A	120.5 (6)	C11B—C10B—C9B	117.6 (6)
C11A—C10A—H10A	119.7	C11B—C10B—H10B	121.2
C9A—C10A—H10A	119.7	C9B—C10B—H10B	121.2
C10A—C11A—C12A	119.4 (6)	C12B—C11B—C10B	121.4 (6)
C10A—C11A—H11A	120.3	C12B—C11B—H11B	119.3
C12A—C11A—H11A	120.3	C10B—C11B—H11B	119.3
C11A—C12A—C6A	120.8 (6)	C11B—C12B—C6B	121.0 (5)
C11A—C12A—H12A	119.6	C11B—C12B—H12B	119.5
C6A—C12A—H12A	119.6	C6B—C12B—H12B	119.5
N1A—C13A—C14A	110.2 (5)	N1B—C13B—C14B	110.8 (4)
N1A—C13A—H13A	109.6	N1B—C13B—H13C	109.5
C14A—C13A—H13A	109.6	C14B—C13B—H13C	109.5
N1A—C13A—H13B	109.6	N1B—C13B—H13D	109.5
C14A—C13A—H13B	109.6	C14B—C13B—H13D	109.5
H13A—C13A—H13B	108.1	H13C—C13B—H13D	108.1
C13A—C14A—C15A	111.9 (5)	C13B—C14B—C15B	111.7 (5)
C13A—C14A—H14A	109.2	C13B—C14B—H14C	109.3
C15A—C14A—H14A	109.2	C15B—C14B—H14C	109.3
C13A—C14A—H14B	109.2	C13B—C14B—H14D	109.3
C15A—C14A—H14B	109.2	C15B—C14B—H14D	109.3
H14A—C14A—H14B	107.9	H14C—C14B—H14D	107.9
O3A—C15A—O2A	123.1 (6)	O3B—C15B—O2B	123.7 (6)
O3A—C15A—C14A	124.0 (6)	O3B—C15B—C14B	122.9 (6)
O2A—C15A—C14A	112.9 (6)	O2B—C15B—C14B	113.3 (6)
			
C1A—N1A—N2A—C4A	−14.3 (8)	C1B—N1B—N2B—C4B	−10.7 (7)
C13A—N1A—N2A—C4A	169.3 (5)	C13B—N1B—N2B—C4B	179.9 (4)
N2A—N1A—C1A—O1A	−176.4 (5)	N2B—N1B—C1B—O1B	−175.2 (5)
C13A—N1A—C1A—O1A	−0.3 (9)	C13B—N1B—C1B—O1B	−6.7 (8)
N2A—N1A—C1A—C2A	2.0 (8)	N2B—N1B—C1B—C2B	3.0 (8)
C13A—N1A—C1A—C2A	178.1 (5)	C13B—N1B—C1B—C2B	171.5 (5)
O1A—C1A—C2A—C3A	−156.2 (7)	O1B—C1B—C2B—C3B	−164.7 (6)
N1A—C1A—C2A—C3A	25.5 (9)	N1B—C1B—C2B—C3B	17.2 (8)
C1A—C2A—C3A—C8A	−173.7 (6)	C1B—C2B—C3B—C8B	−167.9 (6)
C1A—C2A—C3A—C4A	−38.1 (9)	C1B—C2B—C3B—C4B	−28.0 (8)
N1A—N2A—C4A—C5A	−176.8 (5)	N1B—N2B—C4B—C5B	179.7 (4)
N1A—N2A—C4A—C3A	−1.5 (8)	N1B—N2B—C4B—C3B	−3.0 (7)
C2A—C3A—C4A—N2A	26.7 (8)	C8B—C3B—C4B—N2B	164.1 (5)
C8A—C3A—C4A—N2A	166.0 (5)	C2B—C3B—C4B—N2B	21.7 (8)
C2A—C3A—C4A—C5A	−158.1 (6)	C8B—C3B—C4B—C5B	−18.6 (8)
C8A—C3A—C4A—C5A	−18.7 (8)	C2B—C3B—C4B—C5B	−161.0 (5)
N2A—C4A—C5A—C6A	171.5 (5)	N2B—C4B—C5B—C9B	−5.4 (7)
C3A—C4A—C5A—C6A	−3.9 (8)	C3B—C4B—C5B—C9B	177.1 (5)
N2A—C4A—C5A—C9A	−6.1 (8)	N2B—C4B—C5B—C6B	173.8 (5)
C3A—C4A—C5A—C9A	178.6 (5)	C3B—C4B—C5B—C6B	−3.7 (7)
C9A—C5A—C6A—C12A	0.2 (8)	C9B—C5B—C6B—C12B	2.4 (8)
C4A—C5A—C6A—C12A	−177.4 (5)	C4B—C5B—C6B—C12B	−176.8 (5)
C9A—C5A—C6A—C7A	179.9 (5)	C9B—C5B—C6B—C7B	−176.4 (5)
C4A—C5A—C6A—C7A	2.4 (8)	C4B—C5B—C6B—C7B	4.4 (7)
C5A—C6A—C7A—C8A	22.2 (9)	C12B—C6B—C7B—C8B	−162.0 (5)
C12A—C6A—C7A—C8A	−158.0 (6)	C5B—C6B—C7B—C8B	16.8 (8)
C6A—C7A—C8A—C3A	−46.2 (9)	C2B—C3B—C8B—C7B	−178.9 (6)
C2A—C3A—C8A—C7A	179.2 (7)	C4B—C3B—C8B—C7B	41.7 (8)
C4A—C3A—C8A—C7A	44.0 (8)	C6B—C7B—C8B—C3B	−40.6 (8)
C6A—C5A—C9A—C10A	−0.4 (8)	C6B—C5B—C9B—C10B	−2.3 (8)
C4A—C5A—C9A—C10A	177.2 (5)	C4B—C5B—C9B—C10B	176.9 (5)
C5A—C9A—C10A—C11A	0.1 (9)	C5B—C9B—C10B—C11B	1.5 (8)
C9A—C10A—C11A—C12A	0.5 (10)	C9B—C10B—C11B—C12B	−0.8 (9)
C10A—C11A—C12A—C6A	−0.7 (10)	C10B—C11B—C12B—C6B	1.0 (9)
C5A—C6A—C12A—C11A	0.4 (10)	C5B—C6B—C12B—C11B	−1.8 (8)
C7A—C6A—C12A—C11A	−179.4 (6)	C7B—C6B—C12B—C11B	177.0 (5)
C1A—N1A—C13A—C14A	−101.3 (6)	C1B—N1B—C13B—C14B	89.7 (6)
N2A—N1A—C13A—C14A	75.3 (6)	N2B—N1B—C13B—C14B	−100.4 (5)
N1A—C13A—C14A—C15A	−176.6 (5)	N1B—C13B—C14B—C15B	179.8 (4)
C13A—C14A—C15A—O3A	−115.0 (7)	C13B—C14B—C15B—O3B	10.4 (9)
C13A—C14A—C15A—O2A	65.3 (7)	C13B—C14B—C15B—O2B	−170.1 (5)

### Hydrogen-bond geometry (Å, º)

**Table:**

*D*—H···*A*	*D*—H	H···*A*	*D*···*A*	*D*—H···*A*
O2*A*—H2*A*···O1*B*	0.92 (7)	1.78 (7)	2.651 (6)	158 (6)
O2*B*—H2*B*···O1*A*	0.90 (6)	1.75 (6)	2.598 (7)	157 (5)

## References

[bb1] Altomare, A., Cascarano, G., Giacovazzo, C., Guagliardi, A., Burla, M. C., Polidori, G. & Camalli, M. (1994). *J. Appl. Cryst.* **27**, 435.

[bb2] Costantino, L., Rastelli, G., Vescovili, K., Cignarella, G., Vianello, P., Del Corso, A., Cappiello, M., Mura, U. & Barlocco, D. (1996). *J. Med. Chem.* **39**, 4396–4405. 10.1021/jm960124f 8893834

[bb4] Enraf–Nonius (1994). *CAD-4 EXPRESS* Enraf–Nonius, Delft, The Netherlands.

[bb5] Farrugia, L. J. (2012). *J. Appl. Cryst.* **45**, 849–854.

[bb6] Harms, K. & Wocadlo, S. (1995). *XCAD4* University of Marburg, Germany.

[bb7] Masciocchi, D., Gelain, A., Porta, F., Meneghetti, F., Pedretti, A., Celentano, G., Barlocco, D., Legnani, L., Toma, L., Kwon, B.-M., Asai, A. & Villa, S. (2013). *Med. Chem. Commun.* **4**, 1181–1188.

[bb8] Sheldrick, G. M. (2008). *Acta Cryst.* A**64**, 112–122. 10.1107/S0108767307043930 18156677

[bb9] Toma, L., Cignarella, G., Barlocco, D. & Ronchetti, F. (1990). *J. Med. Chem.* **33**, 1591–1594. 10.1021/jm00168a010 2160535

