# Supplementary figures and images for: Crystal structure of 3-(3-oxo-2,3,4,4a,5,6-hexa­hydro­benzo[h]cinnolin-2-yl)propionic acid
Source: Acta Crystallogr Sect E Struct Rep Online. 2014 Sep 6;70(Pt 10):o1088–9. doi: 10.1107/S1600536814019850 (PMC4257230; doi:10.1107/S1600536814019850)

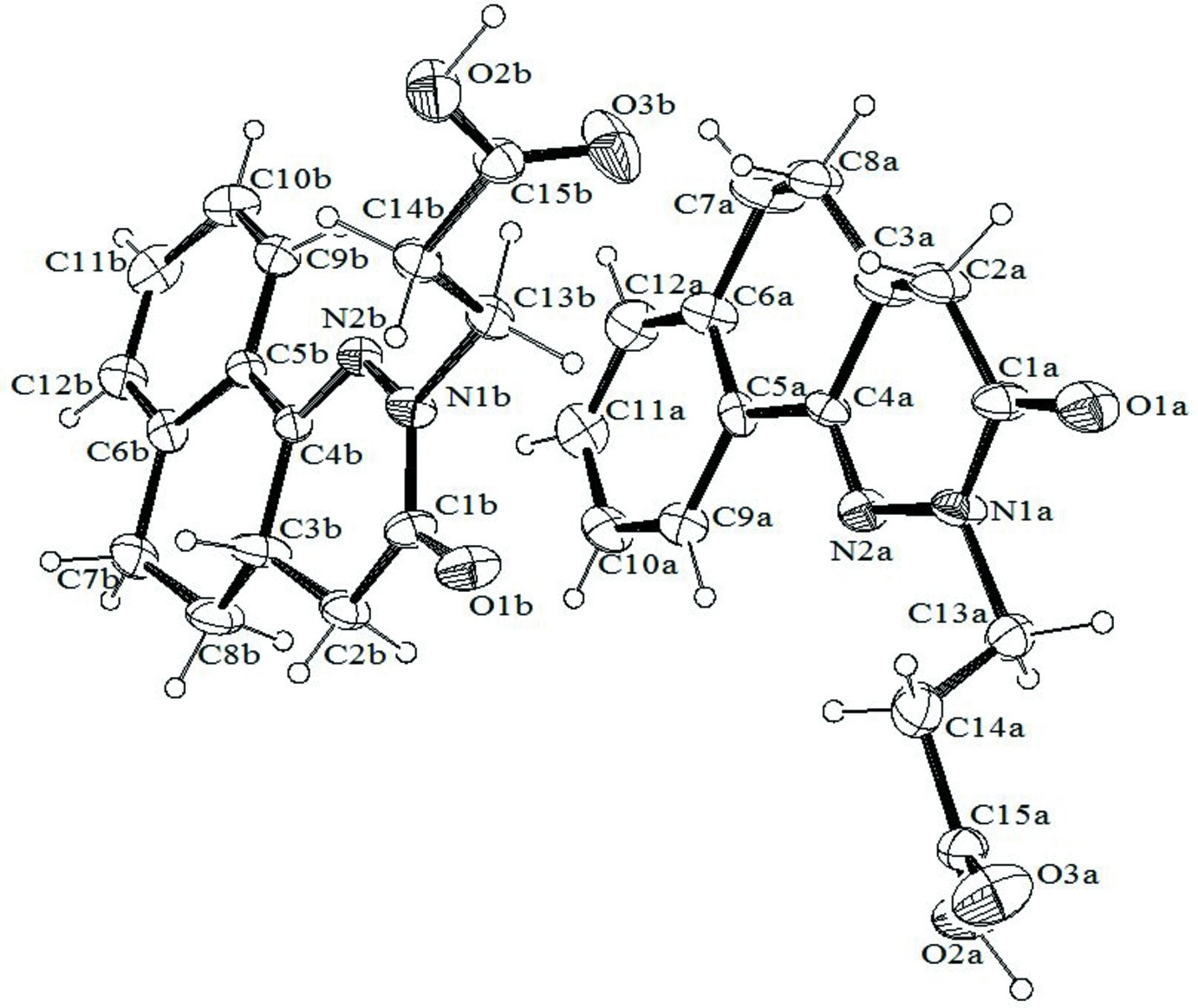

Supplement: Supplementary file 4 [file e-70-o1088-fig1.tif]

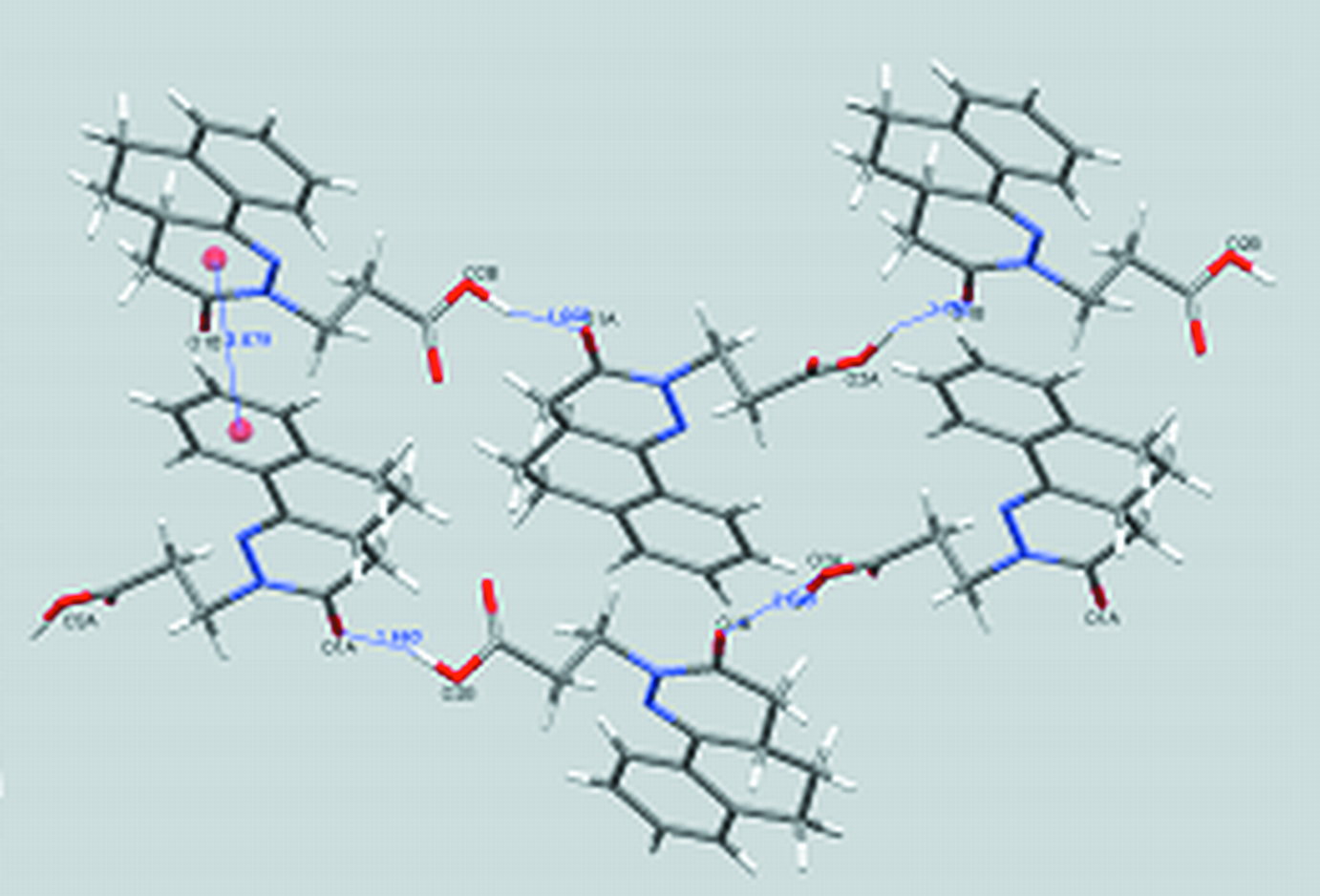

Supplement: Supplementary file 5 [file e-70-o1088-fig2.tif]
